# TAK-242 ameliorates DSS-induced colitis by regulating the gut microbiota and the JAK2/STAT3 signaling pathway

**DOI:** 10.1186/s12934-020-01417-x

**Published:** 2020-08-06

**Authors:** Jiajia Wang, Guannan Zhu, Cheng Sun, Kangwei Xiong, Tingting Yao, Yuan Su, Haiming Fang

**Affiliations:** 1 Department of Pharmacology, School of Basic Medical Sciences, Anhui Medical University, Hefei, Anhui China; 2 Department of Gastroenterology and Hepatology, The Second Hospital of Anhui Medical University, Hefei, Anhui China; 3 Center for Gut Microbiota Research, The Second Hospital of Anhui Medical University, Hefei, Anhui China

**Keywords:** Gut microbiota, Ulcerative colitis, Inflammatory bowel disease, TAK-242, TLR-4, JAK/STAT signaling pathway

## Abstract

**Background:**

The goal of the present study was to investigate the effects of TAK-242 on the gut microbiota and the TLR4/JAK2/STAT3 signaling pathway in mice with dextran sulfate sodium (DSS)-induced colitis.

**Results:**

At the phylum level, *Bacteroidetes*, *Firmicutes*, *Actinobacteria*, *Cyanobacteria*, *Epsilonbacteraeota* and *Proteobacteria* were the primary microbiota in the five groups. TAK-242 treatment significantly enhanced *Verrucomicrobia* and *Actinobacteria*; significantly decreased *Cyanobacteria, Epsilonbacteraeota* and *Proteobacteria*; and particularly promoted the growth of *Akkermansia*. TAK-242 markedly alleviated DSS-induced colitis symptoms and colonic lesions by promoting IL-10 release, inhibiting IL-17 release, downregulating TLR4 and JAK2/STAT3 mRNA and protein expression and increasing JAK2/STAT3 phosphorylation.

**Conclusion:**

TAK-242 modulates the structure of the gut microbiota in colitis and may be a novel therapeutic candidate for ulcerative colitis.

## Background

Ulcerative colitis (UC) is a subtype of inflammatory bowel disease (IBD) characterized by chronic recurrent colonic mucosal inflammation. Epidemiological data have shown that the incidence of IBD, including UC, has been increasing in Western countries and is continuing to rise in developing nations due to the Western diet and lifestyle [1, 2]. Although the etiology and pathogenesis of UC are complicated and still uncertain, genetic predisposition, mucosal immune system deregulation, gut microbiota dysbiosis and environmental factors such as eating habits were demonstrated to be essential factors for the development of UC [3–5]. Current major treatment strategies for UC include aminosalicylic acids, corticosteroids, immunosuppressive agents and biologics, and surgical treatment is required for severe refractory UC. There are still some refractory UC cases due to drug inefficacy, drug dependence, adverse reactions and/or high cost. Therefore, it is extremely important to find new therapeutics for UC.

The gut microbiota coexists with the host, shaping the mucosal and systemic immune systems of the host and acting as a microbial barrier against pathogen invasion. Dysbiosis of the gut microbiota invades intestinal tissue by introduction of invasive antigens into the organism, activation of immune cells, and production of cytokines, resulting in local or systemic inflammation [6]. Additionally, altered gut microbiota compositions have been reported to regulate T-cell differentiation [7]. Gut microbiota dysbiosis is commonly considered to be a key factor in UC. The imbalance of proinflammatory and anti-inflammatory cytokines is also essentially involved in the development of UC associated with excessive immune responses to the abnormal activity of some proinflammatory signals [8]. Regulating the gut microbiota has become a new therapeutic strategy for UC, and our previous research found that reconstruction of gut microbiota via fecal microbiota transplantation (FMT) from healthy donors could be an important therapeutic measure for UC [9]. However, few studies have explored the possible mechanism of gut microbiota dysbiosis and imbalance of the inflammatory response in UC. The link between biogenetics, gut microbes and immune responses is largely unknown.

Inflammation is a defensive response of the body to remove potentially pathogenic stimuli, such as viral, bacterial, or fungal infections, as well as a part of the healing process to repair damaged tissues. Pattern recognition receptors (PRRs) recognize structures that are conserved among microbes and are necessary for the recognition of the presence of microorganisms [10]. Toll-like receptors (TLRs) are of the PRR family and play an essential role in the activation of innate and adaptive immunity systems and defense against invading microbial pathogens and intestinal microbiota [11]. Activation of TLRs can be triggered by pathogens as well as harmful signals from external and internal factors, such as oxidation of stress and inflammatory cytokines derived from tissues or cells [12]. TLR4, one of the TLR families, is expressed at a low concentration in the intestinal mucosa under steady-state conditions and could be activated in response to the lipopolysaccharides (LPSs) produced by gram-negative bacteria [13]. TLR4 knockout mice showed less intestinal inflammation and intestinal barrier disruption [14]. Microbiota dysbiosis can activate the TLR4-mediated inflammatory signaling pathway [15]. TLR4 was upregulated in the colonic tissue of UC patients and enhanced the synthesis and release of inflammatory factors. TLR4-mediated inflammation is an important pathogenic event in UC.

TLR4-directed small molecule pharmacological antagonists have now been identified as a novel class of molecular therapeutics. TLR4 antagonists are in various stages of preclinical and clinical development for the modulation of dysregulated TLR4-dependent inflammatory signaling [16]. Suppressing TLR4-mediated intestinal dysfunction could be used to treat nonalcoholic fatty liver disease, Parkinson's disease, and hepatocellular carcinoma [17–19]. Overexpression of TLR4 plays an important role in the pathogenesis of UC [20]. However, few studies have reported the pharmacological effects of TLR4-directed small molecule antagonists on UC.

TAK-242 (resatorvid) is a novel cyclohexene derivative that selectively binds to TIR domain of TLR4, disrupting its ability to interact with its adaptor molecules and inhibiting TLR4-mediated multiple cytokine production. TAK-242 can suppress the LPS-induced production of TNF-α, IL-1β, IL-6, and NO at IC_50_ values ranging from 11 to 33 nM. In addition, differences in species do not greatly affect the inhibitory effects of TAK-242 on cytokine production. Thus, TAK-242 may be a promising drug for the treatment of inflammatory diseases involving TLR4, such as sepsis [21]. In addition to being able to relieve acute kidney injury, TAK-242 can ameliorate inflammatory injury in cerebral hemorrhage and acute cerebral ischemia/reperfusion, and can also inhibit intimal hyperplasia in murine aortic allografts [22–25]. In addition, TLR4 promotes the production of inflammatory cytokines through MyD88- and TRIF-dependent pathways [26]. In several colitis related studies, TAK-242 has been used as an antagonist to regulate TLR4 expression in vitro and in vivo*,* and the results showed that TAK-242 could block CYP3A4 and P-gp down-regulation induced by bacterial outer-membrane vesicles in Caco-2 cells and inhibit the phenotypic transition of Raw 264.7 macrophages [27, 28]. TAK-242 can also suppress the infiltration of IgG4 + plasma cells, and inhibit the intestinal expression of IgG4 in intestinal in dextran sulfate sodium (DSS) -induced colitis [29]. However, few reports have described the therapeutic potential of TAK-242 in UC to date.

Recent findings have indicated that the Janus kinase/signal transducer and activator of transcription (JAK/STAT) signaling pathway is also involved in the pathogenesis of several diseases, including IBD, and this effect is especially true for JAK inhibitors, which have recently been shown to be effective for the treatment of UC [30]. The JAK family in mammals consists of four JAK members, JAK1, JAK2, JAK3 and tyrosine kinase 2 (TYK 2) [31]. JAKs can phosphorylate other signaling molecules, including STATs, through different cytokine receptors [32]. STATs are a class of potential transcription factors activated by cytokines and growth factors. Mammals have 7 STAT proteins: STAT 1, STAT 2, STAT 3, STAT 4, STAT5A, STAT5B and STAT6. Among them, STAT 3 is a multifunctional member and participates in acute stress reactions, cell growth, differentiation and immune responses [33]. JAK 2/STAT 3 is the major pathway of transcription factors involved in the proinflammatory cytokine response in intestinal mucosal inflammation [34]. In the present research, we investigated the effect of TAK-242 on dextran sulfate sodium (DSS)-induced colitis and analyzed the crosstalk of the gut microbiota and the JAK2/STAT3 signaling pathways to explore whether the TLR4 inhibitor TAK-242 could act as a potential therapeutic option for UC and possible crosstalk mechanism between the gut microbiota and the host. 5-Aminosalicylic acid (5-ASA) is an anti-inflammatory modulator that is the backbone of therapeutic management for mild to moderate UC. Because 5-ASA can affect intestinal bacteria and reduce bacterial invasion and total fecal bacterial abundance [35], we used 5-ASA as the control drug in the present study.

## Results

### TAK-242 alleviates signs and symptoms of DSS-induced colitis

**Fig. 1:**
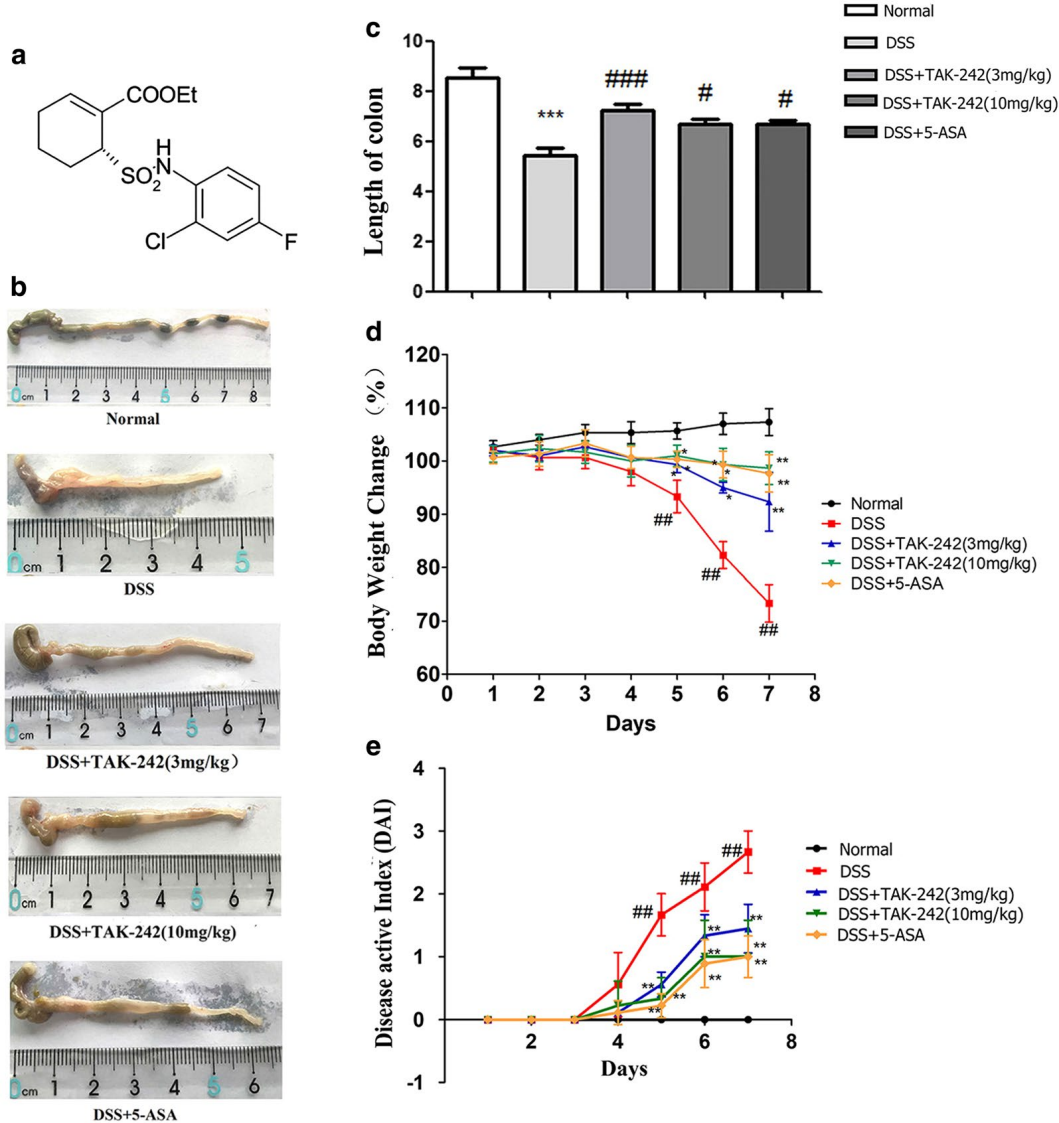
TAK-242 improved the performance of DSS-induced colitis. **a** Molecular structure of TAK 24. **b**, **c** Morphology and length of colon in each group. **d** Body weight change in each group. **e** DAI of each group. The data are expressed as the means ± SEM from ten mice per group and at least three independent experiments. Compared with the control group, DSS group exhibited significant weight loss, diarrhea, colitis manifestations with bloody stool, shortened colon and increased DAI (*p < 0.05, **p < 0.01, ***p < 0.001). Compared with the DSS group, the groups treated with different doses ofTAK-242 had significantly relieved signs and symptoms of DSS-induced colitis (^#^*p* < 0.05, ^##^*p* < 0.01, ^###^*p* < 0.001)

Compared with the control group, mice with DSS-induced colitis exhibited significant weight loss, diarrhea, colitis manifestations with bloody stool, shortened colon and increased DAI. Compared with the model group, the groups treated with different doses of TAK-242 (3 and 10 mg/kg, intraperitoneally administered) had significantly relieved signs and symptoms of DSS-induced colitis (*p* < 0.05). 5-ASA exhibited similar effects as TAK242 in ameliorating the signs and symptoms of DSS-induced colitis (Fig. 1).

### TAK-242 ameliorates colonic lesions of DSS-induced colitis

**Fig. 2:**
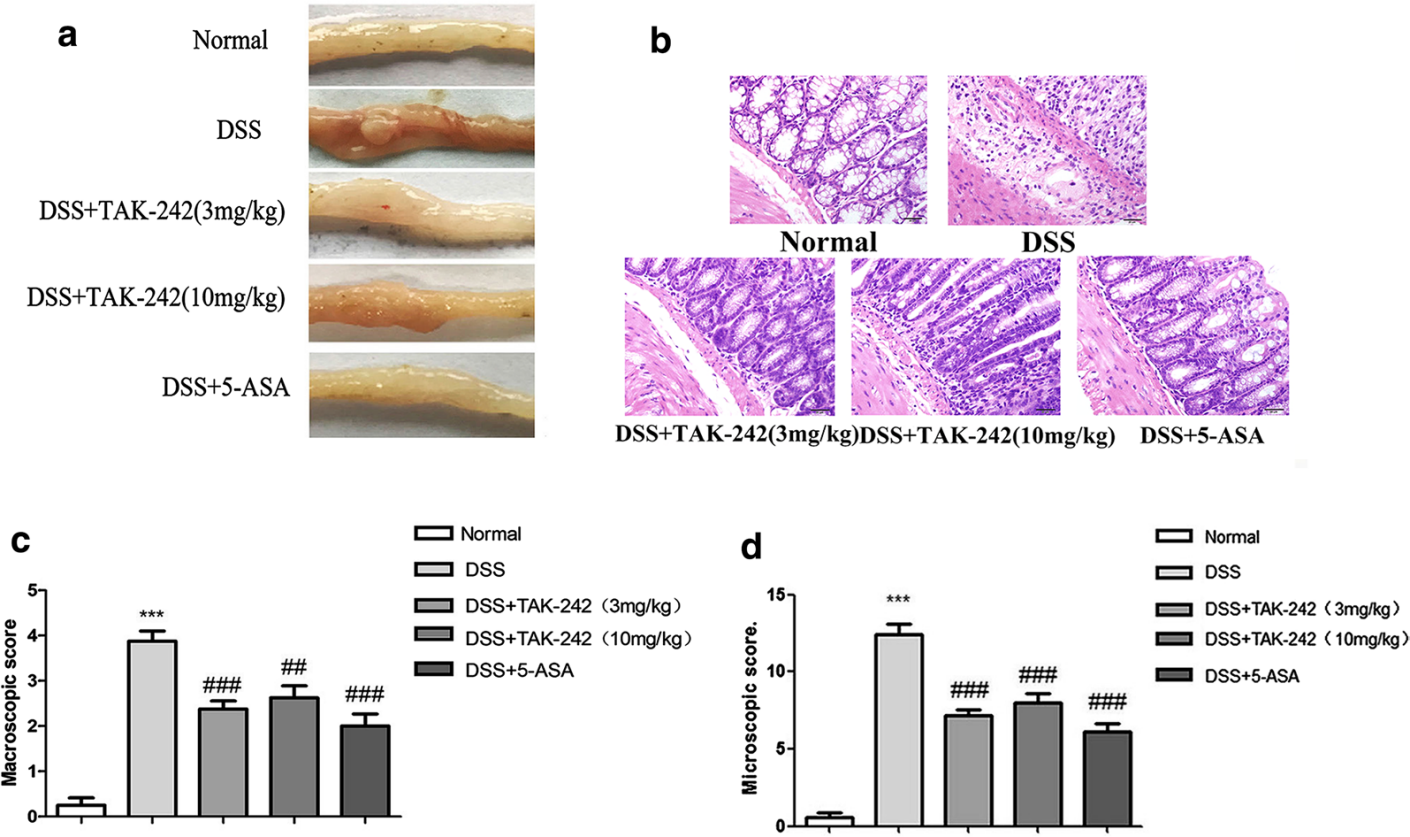
Macroscopic and histopathological assessment of TAK-242 in DSS-induced colitis. **a** Morphology assessment of colon dissection. **b** HE staining of colon sections in each group (400 ×). **c** Macroscopic score of colonic damage in each group. **d** Microscopic score of colonic damage in each group. The data are expressed as the means ± SEM. Compared with the control group, the colonic mucosal lesions in the DSS group were obvious, with a wide range of hyperemia, edema, ulcers and bleeding observed (*p < 0.05, **p < 0.01, ***p < 0.001). Compared with the DSS-induced colitis group, with the treatment of TAK-242, the degree of colonic mucosal lesions was significantly ameliorated ( ^#^*p* < 0.05, ^##^*p* < 0.01, ^###^*p* < 0.001)

After the mice were sacrificed, the entire colon was collected, and the gross morphological damage was first observed. Compared with those in the control group, the colonic mucosal lesions in the DSS group were obvious, with a wide range of hyperemia, edema, ulcers and bleeding observed. Compared with the DSS group, the low-dose TAK-242 group had reduced congestion and bleeding but still had some ulcer bleeding spots, while the high-dose TAK-242 group had significantly reduced congestion, bleeding and ulcers (Fig. 2a and c). Histological evaluation of colon tissue showed that DSS-induced colitis was characterized by epithelial cell destruction, crypt deformation, ulceration, and inflammatory cells infiltrating the lamina propria and submucosa (primarily mononuclear macrophages, neutrophils and eosinophils). With the treatment of different doses of TAK-242, the degree of colonic mucosal lesions was significantly ameliorated, the inflammatory cell infiltration in the mucosa and submucosa was reduced, the colonic mucosa integrity was maintained, and the histological score was decreased (Fig. 2b and d). 5-ASA produced similar effects as TAK242 in improving the colonic lesions associated with DSS-induced colitis.

### TAK-242 regulates the levels of IL-17 and IL-10 in DSS-induced colitis

**Fig. 3:**
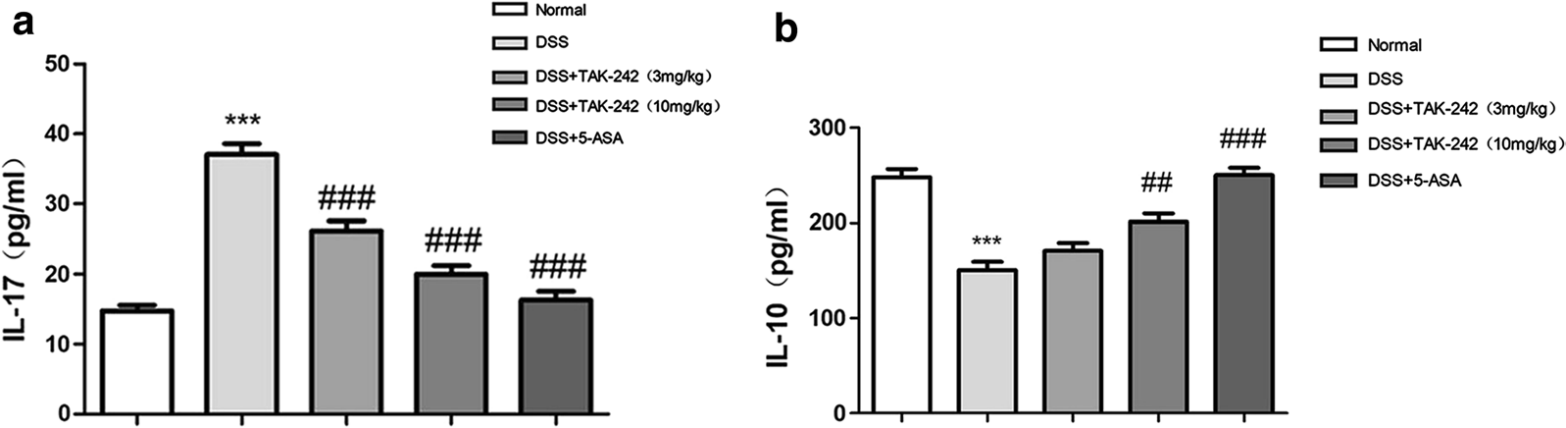
Effects of TAK-242 on the levels of IL-17 (**a**) and IL-10 (**b**) in DSS-induced colitis. The data are expressed as the means ± SEM. Compared with the control group, the DSS group had a significantly enhanced IL-17 levels and a decreased IL-10 levels (*p < 0.05, **p < 0.01, ***p < 0.001). Compared with the DSS group, TAK-242 treatment could decreased serum IL-17 levels and increased IL-10 levels (^#^*p* < 0.05, ^##^*p* < 0.01, ^###^*p* < 0.001)

The levels of interleukin (IL)-10 and IL-17 in plasma are shown in Fig. 3. Compared with that observed in the control group, the DSS group had significantly enhanced IL-17 levels and a decreased IL-10 levels (*p* < 0.05). Compared with the DSS group, the 3 mg/kg and 10 mg/kg TAK-242 groups both had significantly decreased serum IL-17 levels (*p* < 0.05), and the 10 mg/kg TAK-242 group had a significantly increased IL-10 level (*p* < 0.05), while the 3 mg/kg TAK-242 group had an IL-10 level that only slightly increased. It is suggested that TAK-242 may improve damage by reducing proinflammatory factors and increasing anti-inflammatory factors (Fig. 3a, b).

### Effects of TAK-242 on the TLR4/JAK/STAT signaling pathway of colonic tissues in DSS-induced colitis

**Fig. 4:**
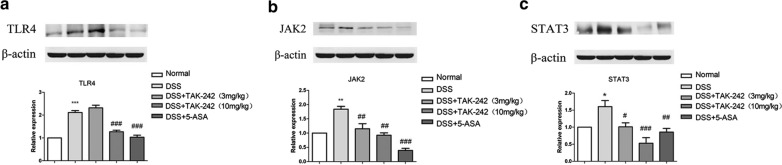
Effects of TAK-242 on the expression of TKR4/JAK2/STAT3 in colitis tissue. The protein expression levels of TLR4, JAK2 and STAT3 were evaluated by western blotting. β-Actin was used as an internal control. The relative band intensity of each protein was measured and compared with that of β-actin. Compared with the control group, the DSS group showed an obvious upregulation of the protein expression of TLR4, JAK2 and STAT3protein expression (*p < 0.05, **p < 0.01, ***p < 0.001).Compared with the DSS group, the group treated with 3 mg/kg TAK-242 exhibited no notable change in TLR4 expression, while the groups treated with 10 mg/kg TAK-242, as well as 5-ASA, exhibited a marked downregulation of TLR4 expression (^#^p < 0.05, ^##^*p* < 0.01, ^###^*p* < 0.001)

**Fig. 5:**
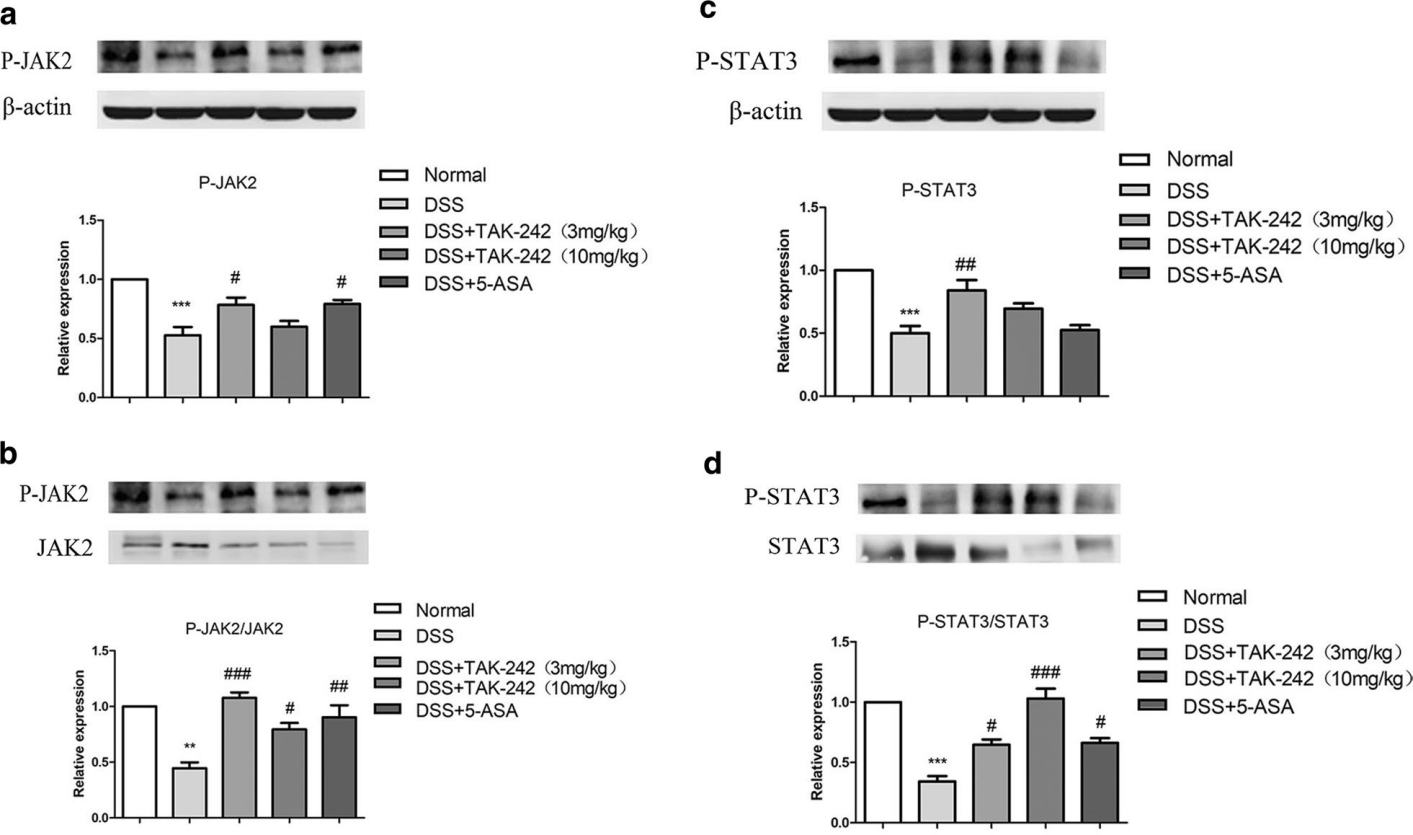
Effects of TAK-242 on the expression of p-JAK2/p-STAT3 in colitis tissue. The protein expression levels of p-JAK2 and p-STAT3 were evaluated by western blotting. β-Actin was used as an internal control. **a** The relative band intensity was measured compared with β-actin. **b** The relative band intensity was measured compared with JAK2 and STAT3. Compared with the control group, the DSS group showed an obvious downregulation of the protein expression of p-JAK2 and p-STAT3 levels (**p < 0.01, ***p < 0.001). Compared with the DSS group, TAK242 significantly enhanced the expression of p-JAK2 and p-STAT3 (^#^*p* < 0.05, ^##^*p* < 0.01, ^###^*p* < 0.001)

**Fig. 6:**
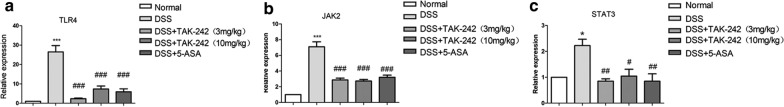
Effects of TAK-242 on the mRNA expression of TLR4/JAK2/STAT3 in colitis tissue. The mRNA levels of TLR4, JAK2 and STAT3 were determined by real-time PCR. Compared with the control group, the DSS group had greatly increased mRNA levels of TLR4, JAK2 and STAT3 (*p < 0.05, ***p < 0.001).Compared with the DSS group, TAK242 treatment significantly decreased the mRNA levels of JAK2 and STAT3 (^#^*p* < 0.05, ^##^*p* < 0.01, *###p* < 0.001)

As the critical role of JAK/STAT activity has been studied in the pathogenesis of UC, the protein levels of TLR4, JAK2, STAT3, phospho-JAK2 and phospho-STAT3 were determined by western blotting (Figs. 4 and 5). The mRNA levels of TLR4, JAK2, and STAT3 were determined by quantitative real-time PCR (qRT-PCR) assays (Fig. 6).

Compared with the control group, mice treated with DSS showed an obvious upregulation of the protein expression of TLR4 in colitis tissue. Compared with the DSS-colitis group, the group treated with 3 mg/kg TAK-242 exhibited no notable change in TLR4 expression, while the groups treated with 10 mg/kg TAK-242, as well as 5-ASA, exhibited a marked downregulation of TLR4 (Fig. 4). JAK2 and STAT3 were upregulated in DSS-induced colitis tissue, while phosphorylated JAK2 and STAT3 were significantly downregulated (Figs. 4 and 5). Compared with the DSS-induced colitis group, the groups treated with TAK-242 had significantly downregulated expression of TLR4. These results indicated that TAK-242 might reduce the expression of JAK2 and STAT3 by inhibiting TLR4. Treatment with different doses of TAK-242 in DSS-induced colitis mice significantly enhanced the expression of phosphorylated JAK2 and STAT3. In summary, DSS induction could activate JAK2, STAT3 translocation and subsequent degradation of phospho-JAK2 and phospho-STAT3, while TAK-242 could ameliorate upregulated JAK2/STAT3 and upregulate phosphorylated JAK2/STAT3.

On the other hand, compared with that observed inwith the control group, the DSS-induced colitis model group had greatly increased mRNA levels of TLR4, JAK2 and STAT3. Furthermore, TAK-242 significantly decreased the mRNA levels of JAK2 and STAT3 (Fig. 6).

### TAK-242 modulates the structure of gut microbiota in DSS-induced colitis mice

**Fig. 7:**
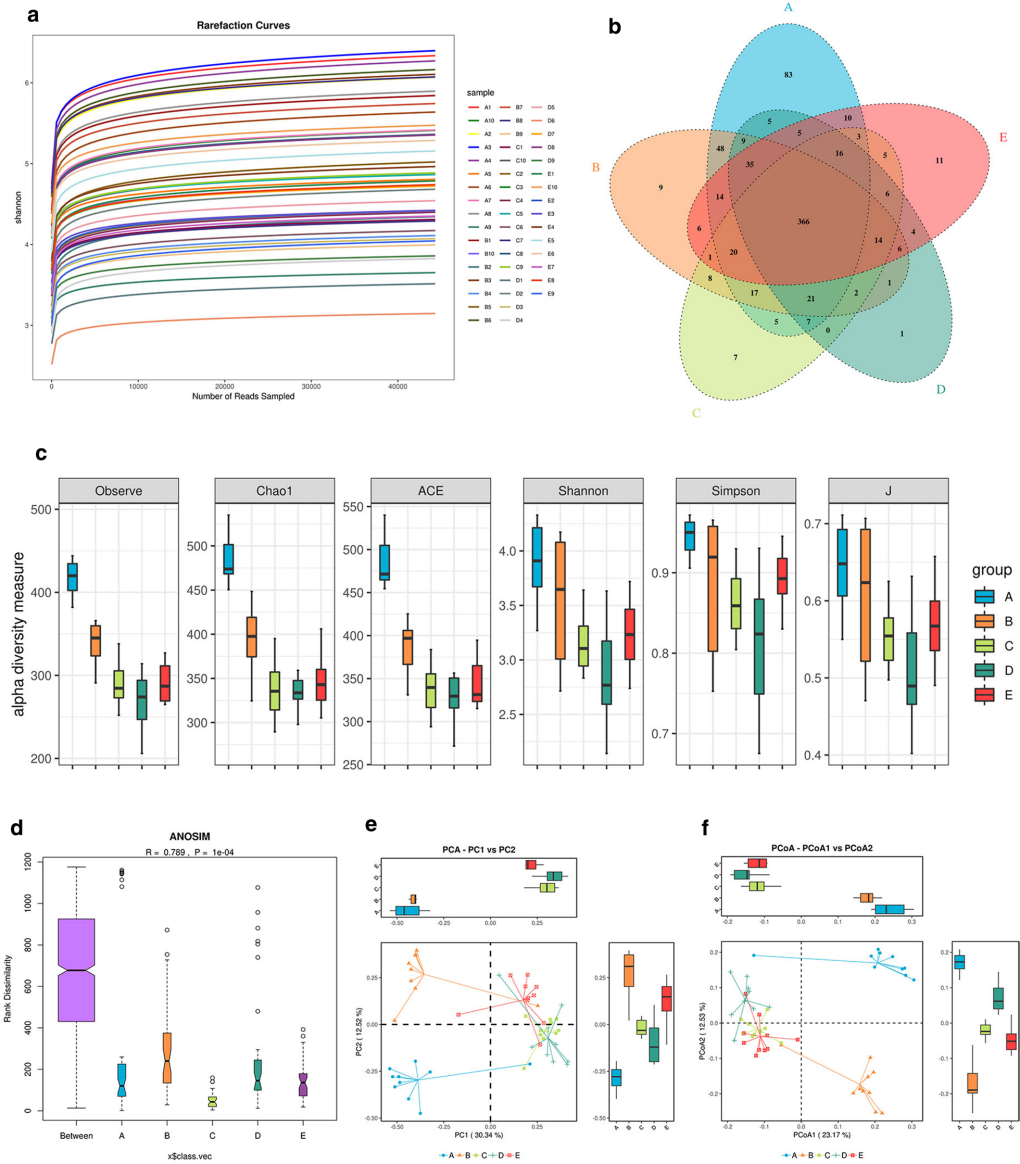
TAK-242 treatment modulated gut microbiota structure in DSS-induced UC mice. **a** Rarefaction curves of all samples. **b** Venn diagram indicating the differential numbers of OTUs in each group. **c** Alpha diversity in each group, as determined by abundance index (Chao1 and ACE) and diversity index (Shannon and Simpson). The alpha diversity index of the fecal microbiota showed significant differences among the groups (*p* < 0.05). **d** Analysis of similarities (ANOSIM) revealed that the differences in the gut microbiota community structure between groups were greater than those within each group. **e** Multiple samples principal component analysis (PCA) in each group. **f** Multiple sample principal coordinate analysis (PCoA) in each group. PCA and PCoA revealed that the gut microbiota in DSS-induced colitis significantly deviated from that observed in the control group. Treatment with TAK-242 markedly improved the distance but did not return the level to the level of the control group. A: Control group, B: DSS group, C: DSS + 3 mg/kg TAK-242 group, D: DSS + 10 mg/kg TAK-242 group, E: DSS + 5-ASA group

The Chao1 and Shannon–Wiener index curves in each sample became smooth. Further more, the sequencing data amount could meet the requirement of the sequencing depth and cover new phylotypes and most of the diversity (Fig. 7a).

The changes in the operational taxonomic units (OTUs) of the five groups are depicted in Venn diagrams. In total, 16,724 OTUs were obtained from 49 samples. The Venn diagram revealed that 366 OTUs coexisted in all five groups; 530 OTUs coexisted between the control and DSS group, 450 OTUs coexisted between the DSS group and the DSS + 3 mg/kg TAK-242 group, 454 OTUs coexisted between the DSS group and the DSS + 10 mg/kg group, 455 OTUs coexisted between the control group and the DSS + 3 mg/kg TAK-242 group, and 464 OTUs coexisted between the control group and the DSS + 10 mg/kg TAK-242 group. The data revealed different OTU diversity in each group (Fig. 7b). The alpha diversity index was calculated by the abundance index (Chao1 and ACE) and diversity index (Shannon and Simpson). The alpha diversity index of the fecal microbiota in the control group showed a significant difference between the DSS groups (Fig. 7c, *p* < 0.05). We also found that the alpha diversity index of the control group was higher than that of the TAK-242 treatment groups, which indicated that TAK-242 treatment could impact the gut microbiota diversity. To measure the level of similarity between gut microbial communities, analysis of similarities (ANOSIM) was performed. The data revealed that the differences in the gut microbiota structure between groups were greater than the differences within each group (Fig. 7d). Principal component analysis (PCA) and principal coordinate analysis (PCoA) revealed that the gut microbiota in DSS-induced colitis significantly deviated from that in the healthy control group. Treatment with TAK-242 markedly improved the distance but did not return the level to the level of the control group. The system clustering tree also indicated a significant difference in the five groups, and the level of the TAK-242 group was close to that of the control group. The effects of 5-ASA were similar to those of TAK-242 (Fig. 7e, f).

### TAK-242 promotes the growth of certain bacteria in DSS-induced colitis mice

According to different diversity indices, DSS reduced the diversity and richness of gut microbiota in stool samples. The linear discriminant analysis (LDA) effect size (LEfSe) analysis method was used to identify biomarkers and dominant microorganisms in each group. At the phylum level, *Bacteroidetes, Firmicutes, Actinobacteria, Epsilonbacteraeota* and *Proteobacteria* were the primary microbiota in the five groups. Compared with that observed in the control group, the relative abundance of *Bacteroidetes* was slightly decreased in the DSS group but significantly decreased in the TAK 242 and 5-ASA groups. The relative abundance of *Firmicutes* showed no significant change among the five groups. Compared with that observed in the control group, the ratio of *Bacteroidetes* to *Firmicutes* was slightly decreased in the DSS group but markedly decreased in the TAK-242 and 5-ASA groups.

**Fig. 8:**
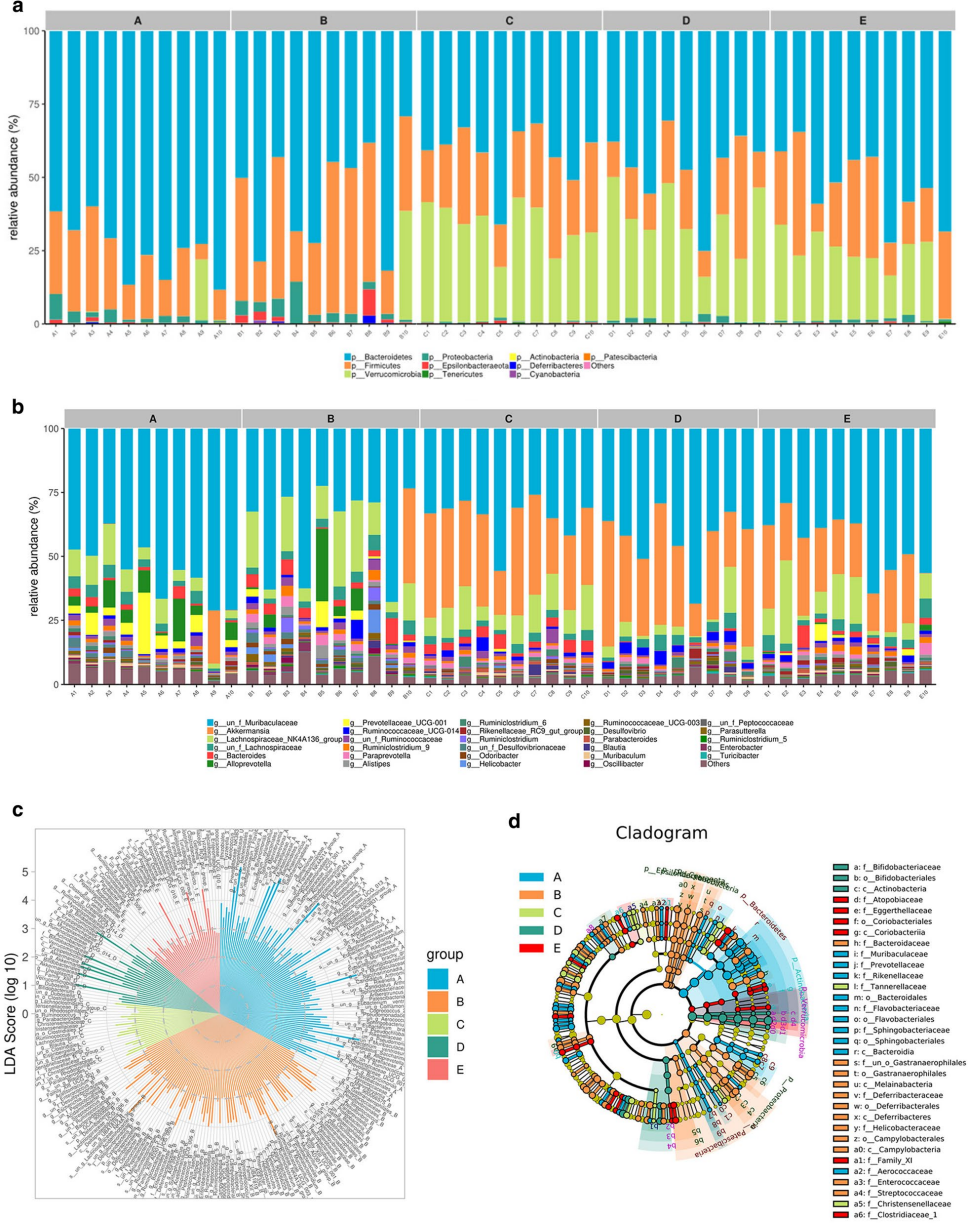
TAK-242 treatment modulated gut microbiota composition at the phylum level in DSS-induced UC mice. **a** Relative abundance of gut microbiota at the phylum level in each group. **b** Relative abundance of gut microbiota at the genera level in each group. **c** Linear discriminant analysis (LDA) score in each group. **d** Cladogram of gut microbiota in each group. Compared with the abundance in the normal group, the abundance of Proteobacteria in DSS-induced mice was markedly higher but was significantly decreased in the TAK-242 groups. Cyanobacteria and Epsilonbacteraeota were significantly increased in the DSS-induced colitis mice, but were obviously decreased after treatment with TAK-242 and 5-ASA. Interestingly, TAK-242 and 5-ASA significantly enhanced the relative abundance of Verrucomicrobia, which was not detectable in the guts of control and DSS-colitis mice. A: Control group, B: DSS group, C: DSS + 3 mg/kg TAK-242 group, D: DSS + 10 mg/kg TAK-242 group, E: DSS + 5-ASA group

**Fig. 9:**
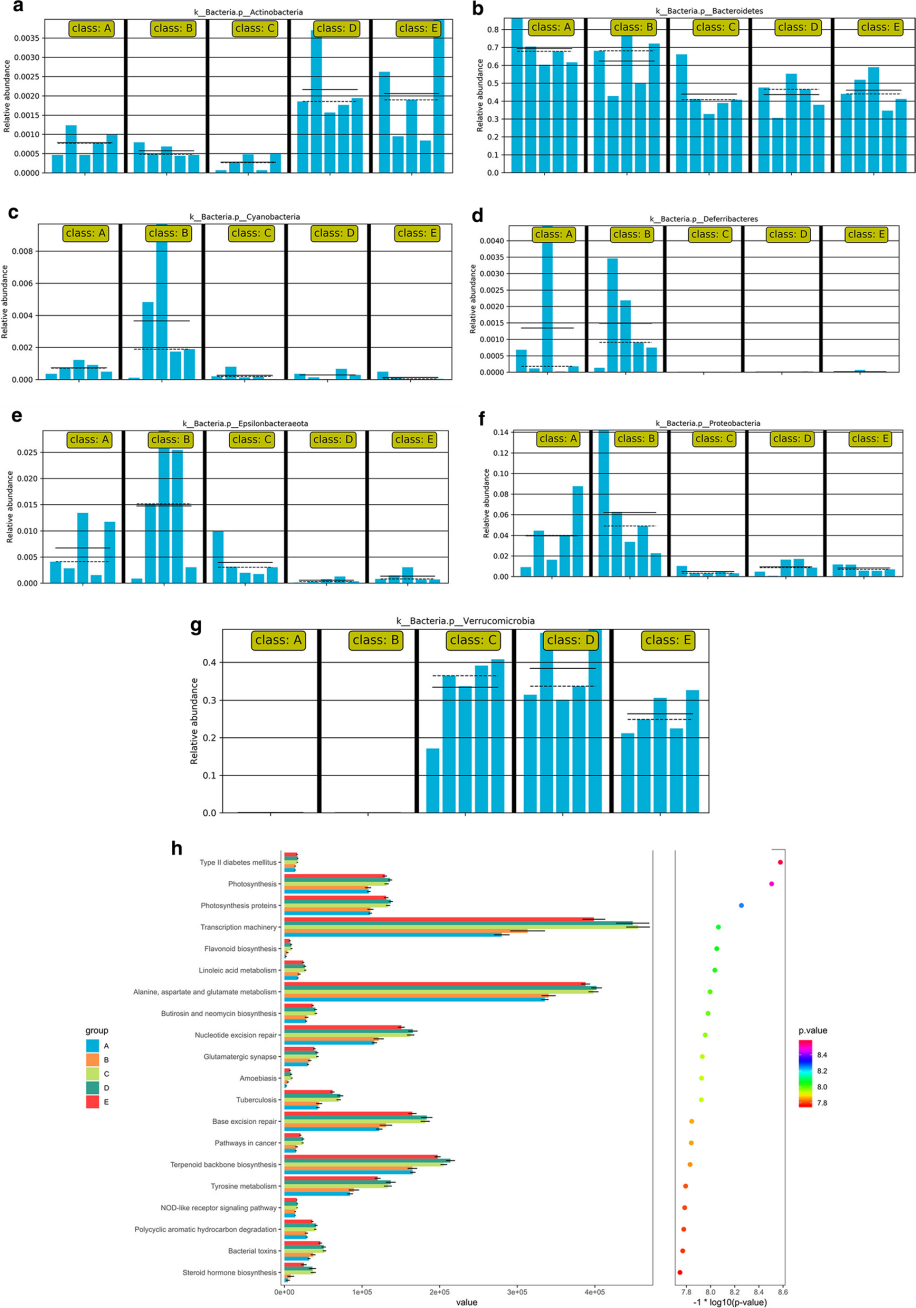
TAK-242 treatment modulated certain gut bacteria in DSS-induced colitis mice. **a** The relative abundance of *Actinobacteria* in each group. **b** The relative abundance of *Bacteroidetes* in each group. **c** The relative abundance of *Cyanobacteria* in each group. **d** The relative abundance of *Deferribacteres* in each group. **e** The relative abundance of *Epsilonbacteraeota* in each group. **f** The relative abundance of *Proteobacteria* in each group. **g** The relative abundance of *Verrucomicrobia* in each group. **h** KEGG pathway functions were categorized using PICRUSt. TAK-242 modulates the structure of gut microbiota in DSS-induced colitis mice (**a**–**g**), PICRUSt predicted analyses found that the gut microbiota pathway functions showed that several pathways in gut microbiome among the four treatments and the control group changed significantly, especially the pathways of transcription machinery, metabolism of alanine, aspartate, and glutamate, nucleotide excision repair, base excision repair, and steroid hormone biosynthesis, respectively (**h**). A: Control group, B: DSS group, C: DSS + 3 mg/kg TAK-242 group, D: DSS + 10 mg/kg TAK-242 group, E: DSS + 5-ASA group

Compared with that observed in the control group, the abundance of *Proteobacteria* in DSS-induced mice was markedly higher but was significantly decreased in the TAK-242 groups. *Cyanobacteria* and *Epsilonbacteraeota* were significantly increased in DSS-induced colitis mice, and they were obviously decreased after treatment with TAK-242 and 5-ASA. Interestingly, TAK-242 and 5-ASA significantly enhanced the relative abundance of *Verrucomicrobia*, which was not detectable in the guts of control and DSS-colitis mice (Fig. 8a–d). Some specific bacteria were identified at the genera level. *Enterobacter*, *Paraprevotella*, *Oscillibacter* and *Ruminiclostridium* showed significantly high relative abundances in DSS-induced colitis mice, and these abundances were significantly decreased by TAK-242. Moreover, TAK-242 significantly promoted the growth of *Akkermansia*. Taken together, these results showed that TAK-242 could regulate the gut microbiota composition in DSS-induced colitis (Fig. 9a–g).

The PICRUSt tool was used to predict the functional profiles of the gut microbiota (Fig. 9h). With the predicted metagenome, Kyoto Encyclopedia of Genes and Genomes (KEGG) pathway functions were categorized using PICRUSt. The gut microbiota pathway functions showed that several pathways in gut microbiome among the four treatment and the control group changed significantly, especially the pathways of transcription machinery; alanine, aspartate, and glutamate metabolism; nucleotide excision repair; base excision repair; and steroid hormone biosynthesis respectively.

## Discussion

The gut microbiota has a major role in health, including in the maturation and education of host immune responses, protection against enteric pathogen proliferation and response to or modification of dysbiosis. Dysbiosis of the gut microbiota affects the intestinal tissue, inducing local or systemic inflammation by the introduction of invasive antigens into the organism, activation of immune cells, and production of cytokines [6]. The pathophysiological characteristics of UC include the cascade of inflammation and the release of multiple proinflammatory and anti-inflammatory cytokines and chemokines to simultaneously fight against stimulation and tissue damage. Although the pathogenesis of UC is largely unknown, the current concept is that it is closely correlated with chronic inflammation, gut barrier disruption and dysbiosis of the gut microbiota. A total of 163 susceptibility loci have been identified in IBD pathogenesis, but these loci only explain a minority of the disease risk variance, indicating that other factors are substantially involved in IBD pathogenesis [36]. The gut microbiota is likely the most crucial environmental factor in IBD pathogenesis; however, the crosstalk mechanisms between the gut microbiota and host still have not been entirely elucidated.

DSS-induced colitis is a well-established and widely accepted model that mimics both the symptoms and morphological changes in human UC. In the present study, mice with DSS-induced colitis exhibited dysbiosis and disrupted metabolic activity of the gut microbiota. DSS induced upregulation of *Proteobacteria, Cyanobacteria, Deferribacteres,* and *Deferribacteres. Proteobacteria* are considered the primary pathogenic bacteria, and *Proteobacteria* can produce endotoxins [37, 38]. The increased abundance of *Proteobacteria* may be correlated with the inflammatory response to DSS. Treatment of mice with DSS colitis with TAK-242 markedly relieved diarrhea and decreased DAI, which reflected the severity of weight loss, stool consistency and blood in stool. TAK-242 ameliorated DSS-induced colonic lesions in a dose-dependent manner.

Analysis of the fecal microbiota showed that DSS treatment could reduce the diversity of the gut microbiota community. The beta diversity of PCA and PCoA revealed that the gut microbiota in the DSS-induced colitis group significantly deviated from that in the control group, which was consistent with a previous report [39]. Dysbiosis and dysfunction of the gut microbiota in DSS-induced colitis mice were regulated by TAK-242 treatment. TAK-242 significantly downregulated the increased abundance of *Proteobacteria.* The relative abundance of *Firmicutes* showed no significant change after treatment with TAK-242. Interestingly, TAK-242 significantly enhanced the relative abundance of *Verrucomicrobia* and significantly promoted the relative abundance of *Akkermansia*. These results indicated that TAK-242 could regulate the microbial composition in the intestine and enhance the proliferation of certain bacteria, such as *Akkermansia*.

*Akkermansia muciniphila* was isolated from a human fecal sample a decade ago. Its specialization in mucin degradation makes it a key organism at the mucosal interface between the lumen and host cells. *Akkermansia muciniphila* is representative of the phylum *Verrucomicrobia*. *Akkermansia muciniphila *is associated with healthy mucosa and tends to widely colonize the nutrient-rich intestinal mucus layer and exerts a major role in maintaining gut barrier function, host metabolism and other host biological functions via interactions between the gut microbiota and the host [40, 41]. Reduced levels of *Akkermansia muciniphila* have been observed in patients with IBD, especially UC and metabolic disorders, suggesting that *Akkermansia muciniphila* may have potential anti-inflammatory properties [42]. *Akkermansia muciniphila* could ameliorate DSS-induced UC by regulating some critical inflammatory response components, including pro-inflammatory cytokines (TNF-α, IL-6, IL-12A, INF-γ, and IL-1α), anti-inflammatory cytokines (IL-10), and chemokines (MIP-1α, G-CSF, and KC) [43]. The specific protein Amuc_1100, isolated from the outer membrane of *Akkermansia muciniphila,* could interact with TLR-2 and improve the gut barrier and partly recapitulate the beneficial effects of the bacterium [44].

Metabolic pathways of gut microbiota have been associated with UC disease severity [45]. The results of our PICRUSt predicted analyses found that the gut microbiota pathway functions showed that several pathways in gut microbiome among the four treatments and the control group changed significantly, especially the pathways of transcription machinery, metabolism of alanine, aspartate, and glutamate, nucleotide excision repair, base excision repair, and steroid hormone biosynthesis, respectively. This finding suggested that TAK-242 could also impact gut microbiota metabolism, which may be related with reducing inflammation. All the above results indicated that TLR4 might be an important regulator in the crosstalk between the gut microbiota and the host in UC. Inhibition of TLR4 via a specific inhibitor, TAK-242, could regulate the dysbiosis of gut microbiota and partly recover the levels of beneficial bacteria such as *Akkermansia.*

IL-10 has been critically linked to the prevention of colitis, as mice deficient in IL-10 or IL-10 receptor-chain 1 spontaneously develop colitis [46]. IL-10 could inhibit the expression of proinflammatory mediators such as cell surface receptors, chemokines, and cytokines. IL-17 is a potent pro-inflammatory cytokine that is also considered to be an important mediator in the pathogenesis of intestinal inflammation. IL-17-producing Th17 cells are often present at sites of chronic tissue inflammation in multiple autoimmune diseases in which they function as important drivers of inflammation [47]. Additionally, IL-10 is also involved in the regulation of Th17 cell differentiation, which participates in inflammatory responses by secreting IL-17. IL-10 and IL-17 have coordinating effects in regulating the inflammatory response and maintaining immune homeostasis [48]. Disruption of the Th17/Treg balance is an important factor in IBD pathogenesis and blocking the Th17 axis, either by directly inhibiting IL-17 directly or via preventing Th17 cell differentiation,is currently an area of intense therapeutic development [49].

In the present study, compared with that observed in the control group, the DSS group had a decreased IL-10 levels and a significantly increased IL-17 levels. TAK-242 pretreatment exerted protective effects by regulating the secretion of anti-inflammatory cytokines and proinflammatory cytokines, which were embodied in the increase in IL-10 and decrease in IL-17 levels. On the other hand, IL-10 is also one of the key molecules participating in the regulation of gut homeostasis [50]. In addition, IL-17-producing Th17 cells are likely to be induced by specific components of gut microbiota [51] We found that TAK-242 significantly increased the relative abundance of *Akkermansia*. As mentioned above, *Akkermansia* could ameliorate DSS-induced UC by regulating some critical inflammatory response components, including pro-inflammatory cytokines and anti-inflammatory cytokines. We speculate that *Akkermansia* or other bacteria may be involved in the protective effects of TAK-242 against colitis. Further studies need to be performed to confirm whether the effects of TAK-242 on colitis are due to the regulation of *Proteobacteria* and/or *Akkermansia.*

The JAK2/STAT3 pathway plays an important role in inflammation, which is a positive immune regulator and might play a critical role in regulating the gut microbiome and in maintaining intestinal homeostasis [52]. STAT3 is overexpressed in the colonic mucosa of patients with active and inactive IBD [53]. Tofacitinib, a pan-JAK inhibitor, has been developed as a synergistic anticytokine therapy in IBD, and novel subtype-selective JAK inhibitors are currently being developed and tested as potential novel therapies [30]. To clarify the underlying mechanism of TAK-242 on DSS-induced colitis, proteins and mRNA of the JAK2/STAT3 signaling pathway were detected by western blotting and real-time PCR. Compared with the control group, the DSS-induced colitis group had significantly enhanced gene and protein expression of TLR4. TAK-242 markedly reversed the increased TLR4 expression. The proteins and mRNA of JAK2/STAT3 were significantly upregulated, while phosphorylation of JAK2 and STAT3 was downregulated in DSS-induced colitis mice. Pretreatment with TAK-242 significantly reduced JAK2/STAT3 protein expression and promoted the phosphorylation of JAK2/STAT3, exerting protective effects on colitis. Some data showed similar findings. The expression of p-STAT3 was significantly decreased in colitis, and berberine hydrochloride had a protective effect by inducing the expression of p-STAT3 [54, 55]. Although this was not consistent with the mainstream finding regarding the STAT3 signaling pathway in UC, STAT3 would be activated and p-STAT3 would be upregulated in colitis tissues. We speculate that this may be related to the complex role of STAT3, as it has a dual function: a proinflammatory function in effector Th cells but an anti-inflammatory function in Treg cells. There is a significant overlap between the JAK/STAT signaling of different cytokines. STATs could be activated by other upstream signaling molecules, and JAKs could phosphorylate other downstream targets. The type of activated STATs in the signaling pathway primarily depends on the type of cell and the specific cytokine that initiates signaling. This suggests the complex role of cytokines in regulating the communication between gut microbiota and host immunity. The enhanced level of IL-10 and decreased level of IL-17caused by TAK-242 treatment and/or an increased abundance of *Akkermansia* or other microbiota may be involved in the regulation of the JAK2/STAT3 signaling pathway. Metagenomic sequencing can be used to accurately identify constituents and should promote further research on the strains corresponding to the protective effects of TAK-242 against colitis at the genetic and functional levels.

In summary, TAK-242 had a potential protective effect on DSS-induced colitis and may be expected to be a potential candidate for UC. The JAK2/STAT3 signaling pathway may be involved in this regulation. However, there is a significant overlap between the JAK/STAT signaling of different cytokines. STATs could be activated by other upstream signaling molecules, and JAKs could phosphorylate other downstream target genes. According to a literature search, this is the first report of the therapeutic potential of TAK-242 in UC, and we obtained some interesting preliminary findings. Another important TLR4 signaling pathway, the TLR4/NF-κB signaling pathway, plays an important role in regulating the production of inflammatory cytokines. Further research is needed to confirm these findings.

## Conclusion

Our study demonstrates for the first time that TAK-242 could ameliorate DSS-induced colonic lesions, partially by regulating the JAK2/STAT3 signaling pathway and modulating the structure of the gut microbiota. TAK-242 could promote the growth of certain gut bacteria, such as *Akkermansia* and may be a novel therapeutic candidate for UC. Some specific experiments, such as blocking JAK2/STAT3 signaling, are still needed to obtain more evidence in the future.

## Materials and methods

### Animals and reagents

Six- to eight-week-old male C57BL/6 mice were purchased from the Animal Center of Anhui Medical University and caged under controlled conditions [light (12-h light/dark cycle), humidity (50% ± 5%), and temperature (23 ± 2 °C)]. All mice adapted to the laboratory conditions for 1 week prior to the experiment. DSS (molecular weight 36,000–50,000 kDa) and 5-ASA were purchased from Dalian Meilun Biological Technology Co., Ltd.

TAK-242 powder [resatorvid, ethyl(6R)-6-[N-(2-chloro-4-fluorophenyl)sulfamoyl]cyclohex-1-ene-1-carboxylate] was purchased from Med Chem Express (MCE, NO.10568) and stored at − 20 °C for subsequen use. Enzyme-linked immunosorbent assay (ELISA) kits for mouse IL-10 (NO. CK-E20005M) and IL-17 (NO.CK-E30480M) were purchased from Calvin Biotechnology Co. (Suzhou, China). Electrochemiluminescence (ECL) western blot detection reagents were from Bridgen (Beijing, China). The Ethics Committee of Experimental Animals of Anhui Medical University approved the animal trials (NO. LLSC20170356). All protocols were conducted in accordance with the Administrative Measures of Experimental Animals in Anhui Province and supervised by the Administration Committee of Experimental Animals in Anhui Province.

### Induction of colitis and treatment

Fifty male C57BL/6 mice were randomly divided into five groups with ten mice per group (A group = control control, B group = DSS-induced colitis model, C group = DSS + TAK-242 3 mg/kg, D group = DSS + TAK-242 10 mg/kg, E group = DSS + 5-ASA group). Colitis was induced in mice by administrating 3% DSS in distilled water continuously for 7 days and the control group received the same distilled water without DSS. TAK-242 was dissolved in 1% DMSO and intraperitoneally administered (3 or 10 mg/kg) in an application volume of 0.1 mL/10 g body weight every other day for 7 days. 5-ASA (500 mg/kg) was dissolved in 1.5% carboxymethylcellulose sodium and administered by gavage in an application volume of 0.1 mL/10 g body weight once daily for 7 days. During the experiment, body weight, stool morphology and the presence of gross blood in feces and anus were observed daily. After 7 days, blood was collected through the retro-orbital vein, and serum was obtained by centrifugation at 5000 × *g* for 15 min and stored at − 80 °C prior to the analysis. Then, the animals were sacrificed, and the colons were excised. The length from the cecum to anus was measured. Stool samples in colons were freshly collected, placed in sterile tubes and stored at − 80 °C for microbiota analysis.

### The disease activity index (DAI)

DAI values were was determined by scoring the extent of body weight loss, stool hemoccult positivity or gross bleeding, and stool consistency in accordance with a previously described method (Table 1). DAI = (combined score of weight loss, stool consistency and bleeding)/3 [56]. The DAI scoring was determined each day from day 1 to day 7.

**Table 1:** Disease activity index

Score	Weight loss (%)	Stool consistency*	Occult/gross bleeding
0	(−)	Normal	Normal
1	1–5		
2	6–10	Loose	Guaiac ( +)
3	11–15		
4	> 15	Diarrhea	Gross bleeding

*Normal stools = well-formed pellets; loose = pasty stools which do not stick to the anus; diarrhea = liquid stools that stick to the anus.

### Macroscopic and histological evaluation of colitis

The colon was removed from sacrificed mice, and the length was measured. Subsequently, the intestine was washed with ice-cold phosphate-buffered saline (PBS, pH 7.4) and opened longitudinally. Macroscopic lesions were assessed using the colonic macroscopic scoring system as previously described [57]: 0, no ulcer, no inflammation; 1, no ulcer, local hyperemia; 2, ulceration without hyperemia; 3, ulceration and inflammation at one site only; 4, 2 or more sites of ulceration and inflammation; and 5, ulceration extending more than 2 cm.

The colons were excised, and the distal colonic tissues were fixed in 4% paraformaldehyde, embedded in paraffin, sectioned, and stained with hematoxylin and eosin (H&E). Histological analysis was assessed according to a previously established scoring system to evaluate the severity of the pathological change [58]. Every section was graded by crypt damage (0 = none, 1 = basal 1/3 damage, 2 = basal 2/3 damage, 3 = crypt lost, surface epithelium present, and 4 = crypt and surface epithelium lost), depth of inflammation (0 = none, 1 = mucosa, 2 = submucosa, and 3 = transmural), injury extent (0 = none, 1 = 1–25%, 2 = 26–50%, 3 = 51–75%, and 4 = 76–100%) and inflammatory cell infiltration (0 = none, 1 = light, 2 = medium, and 3 = severe). The researchers performing the scoring were blinded to the treatment conditions.

### ELISA

Blood was collected into a vacuum blood tube containing heparin. Plasma was isolated by centrifuging the blood at 12,000 r/min for 15 min and then stored at − 80 °C. The serum levels of IL-10 (Suzhou, China, NO. CK-E20005M) and IL-17 (Suzhou, China, NO.CK-E30480M) were measured using ELISA kits according to the manufacturer’s instructions.

### Western blotting

Proteins from the colon samples were extracted in lysis buffer containing protease and phosphatase inhibitors. Protein concentrations (n = 5) were determined in the supernatant of colonic tissues by a classic BCA assay kit (Beyotime, China). Equal amounts of proteins (30 μg) were fractionated by 10% SDS-PAGE gels and transferred onto a polyvinylidene fluoride (PVDF; Millipore, USA) membrane by a Bio-Rad western blot apparatus. The membranes were blocked with 5% BSA for 1 h at room temperature and then incubated with the following primary antibodies for 24 h at 4 °C: rabbit anti-JAK2 (1:5000, Abcam, UK), rabbit anti-phospho-JAK2 (1:500–2000, Bioss, China), rabbit anti-TLR4 (1:500–1:2000, Bioss, China), mouse anti-STAT3 (1:500–1000, Bioss, China), rabbit anti-phospho-STAT3 (1:500–2000, Bioss, China), and mouse anti-β-actin (1:1000, ZSGB-BIO, China). After washing with TBST (Tris-buffered solution, pH 7.6, 0.05% Tween 20) 3 times for 10 min, the blots were incubated with anti-rabbit or anti-mouse IgG horseradish secondary antibodies (1/100,000 dilution) for 1 h at room temperature. Finally, the protein bands were visualized with ECL western blot detection reagents (Bridgen, China). The expression levels of the proteins were compared with the control based on the relative intensities of the bands.

### Quantitative real-time PCR

Real-time quantitative polymerase chain reaction was also used to detect the expression of TLR4, JAK2 and STAT3 in colonic tissue. Total RNA was isolated from the colonic tissue using TRIzol reagent (Invitrogen, Carlsbad, CA), washed with 75% ethanol, dissolved in DEPC and kept at − 80 °C. Total RNA (2 µg) was reverse transcribed into cDNA. Quantitative real-time PCR was performed by using a 7500 Real-Time PCR System (Applied Biosystems, Foster City, CA, USA) with SYBR® premix Ex Taq (TaKaRa, Cambridge, MA, USA) according to the manufacturer’s instructions. Targeted cDNAs were amplified at 95 °C for 30 s (1 cycle), 95 °C for 5 s and 60 °C for 34 s (40 cycles). The primer sequences of each target gene were as follows: TLR4 (forward 5′-CGCTTTCACCTCTGCCTTCACTACAG-3′, and reverse 5′-ACACTACCACAATAACCTTCCGGCTC-3′), STAT3 (forward 5′-GTTCCTGGCACCTTGGATTGAGAG-3′, and reverse 5′-CTGTCACTACGGCGGCTGTTG-3′), JAK2 (forward 5′-GTGGAGATGTGCCGCTATG-3′, and reverse 5′-CCTTGTACTTCACGATGTTGTC-3′), and β-actin (forward 5′-TCCTCCTGAGCGCAAGTACTCT-3′, and reverse 5′-GCTCAGTAACAGTCCGCCTAGAA-3′). β-Actin was used as an internal control. The data were analyzed using the comparative threshold cycle (^ΔΔ^Ct) method, 2^−ΔΔCt^ = [(Ct gene of interest—Ct internal control) sample A- (Ct gene of interest—Ct internal control) sample B] according to a previous study [59]

### Fecal microbiota 16S rRNA analysis

DNA was extracted from approximately 0.25 g of fecal samples using the QIAamp Fast DNA Stool Mini Kit (Qiagen, CA, USA) according to the manufacturer’s instructions. The concentrations and purity of the isolated DNAs were assessed using spectrophotometry (Multiskan™ GO, Thermo Fisher Scientific, USA). The DNA extracts were also evaluated for quality using agarose (1.5%) gel electrophoresis in 1 × Tris–acetate-EDTA buffer. DNA samples were stored at − 20 °C before being used as templates for next-generation sequencing library preparation.

The V3-V4 hypervariable region of the bacterial 16S ribosomal RNA (rRNA) gene was amplified from the DNA samples with the barcoded forward primer 515F (5′-GTGCCAGCMGCCGCGGTAA-3′) and the reverse primer 806R (5′-GGACTACNVGGGTWTCTAAT-3′) using KAPA HiFi Hot Start Ready Mix (KAPA Biosystems, USA). Thermal cycling consisted of initial denaturation at 95 °C for 3 min, followed by 30 cycles of denaturation at 95 °C for 20 s, annealing at 60 °C for 30 s, elongation at 72 °C for 30 s and 72 °C for 10 min. PCR products were mixed in equidensity ratios. Then, the PCR product mixture was purified with a Gene JET Gel Extraction Kit (Thermo Scientific). Sequencing libraries were generated using the TruSeq® DNA PCR-Free Sample Preparation Kit (Illumina) following the manufacturer’s recommendations, and index codes were added. The library quality was assessed on the Qubit 2.0 Fluorometer (Thermo Scientific) and Agilent Bioanalyzer 2100 system. Finally, the library was sequenced on an Illumina MiniSeq, and 150 bp paired-end reads were generated.

The raw paired-end reads were assembled using Flash (version 1.2.11). The chimera checking and operational taxonomic unit (OTU) clustering were performed with the clean tags by Usearch 10, following the pipeline. In detail, all reads were demultiplexed into one file, clustered at 97% similarity, and then chimera checking was performed using UCHIME in reference mode. Representative sequences were generated, singletons were removed, and a final OTU table was created. The representative sequences of OTUs were aligned on the Greengenes database for taxonomic classification by RDP Classifier. The phylogenetic trees were produced using FastTree.

### Statistical analysis

Estimates of alpha diversity were based on an evenly rarefied OTU abundance matrix and included observed richness, observed species, Shannon index, Simpson index, ACE, Chao1 and Pielou’s evenness (J’), using the R package vegan (version 2.5-6). The significance difference in measured α-diversity metrics across samples was tested using a nonparametric Kruskal–Wallis rank sum test and Benjamini-Hochbery corrections. The β-diversity of the samples was measured using Bray–Curtis distance based on an evenly rarefied OTU abundance table. The β-diversity can estimate the difference in community structure between samples. Statistical differences in measured β-diversity metrics across groups were determined using PERMANOVA with 999 permutations and adonis in the R package vegan. Shared OTUs were calculated and visualized using the R package Venn Diagram (version 1.6.20). The taxa abundance was measured and plotted using ggplot2 (version 3.2.1). The LEfSe analysis was performed to identify taxa with different abundances in different groups. LEfSe is an algorithm for high-dimensional biomarker discovery and an explanation that identifies genomic features characterizing the differences between two of more biological conditions. Moreover, indicator analysis based on genera was conducted using the R package indicspecies (version 1.7.8). Indicator taxa analysis is a way to calculate the probability that any taxon is found in different groups. A taxon with a high indicator value has a high probability of being found within a given treatment and a low probability of being found outside the treatment, and the p-values were corrected with the method of Benjamini-Hochbery using the p.adjust function in R. Finally, the results were visualized using the custom R script based on ggplot2. These analyses were performed using R (version 3.5.0).

To predict metagenomic functional contents, the Phylogenetic Investigation of Communities by Reconstruction of Unobserved States (PICRUSt) was used to predict which genes were present using the 16S data. The software utilizes a computational approach to predict the functional pathway from the 16S rDNA reads. First, the reads were against a reference collection, the Greengenes database, May 2013 version, and the closed-reference OTU table was built using QIIME (version 1.9.0). The resulting OTU table was normalized by normalize_by_copy_number.py script, and metagenome predictions were conducted using predict_metagenomes.py script. The significant difference analysis was determined using ANOVA. The results were visualized using custom R script based on ggplot2 (version 3.2.1).

Data are expressed as the means ± SDs unless otherwise indicated. Statistical significance was evaluated using SPSS 13.0 by one-way ANOVA. *P* < 0.05 was considered statistically significant.

## Data Availability

The datasets generated and/or analyzed during the present study are available in the [PRJNA615851] repository, [https://submit.ncbi.nlm.nih.gov/subs/bioproject/]

## Abbreviations

UC: Ulcerative colitis
IBD: Inflammatory bowel disease
FMT: Fecal microbiota transplantation
PRRs: Pattern recognition receptors
TLRs: Toll-like receptors
LPSs: Lipopolysaccharides
JAK/STAT: Janus kinase/signal transducer and activator of transcription
DSS: Dextran sulfate sodium
5-ASA: 5-aminosalicylic acid
DAI: Disease activity index
OTU: Operational taxonomic unit
H&E: Hematoxylin and eosin
ANOSIM: Analysis of similarities
PCA: Principal component analysis
PCoA: Principal coordinate analysis
LEfSe: Linear discriminant analysis (LDA) effect size
